# Exploring the health consequences of armed conflict: the perspective of Northeast Ethiopia, 2022: a qualitative study

**DOI:** 10.1186/s12889-023-16983-z

**Published:** 2023-10-24

**Authors:** Mulugeta Wodaje Arage, Henok Kumsa, Mulu Shiferaw Asfaw, Abebe Tarekegn Kassaw, Ephrem Mebratu Dagnew, Abayneh Tunta, Woldeteklehymanot Kassahun, Amanuel Addisu, Molla Yigzaw, Tilahun Hailu, Lebeza Alemu Tenaw

**Affiliations:** 1 School of Midwifery, College of Health Sciences, Woldia University, Woldia, Ethiopia; 2 School of Medicine, College of Health Sciences, Woldia University, Woldia, Ethiopia; 3 Department of Pharmacy, College of Health Sciences, Woldia University, Woldia, Ethiopia; 4 Department of Pharmacy, College of Medicine and Health Sciences, Debre Markos University, Debre Markos, Ethiopia; 5 Department of Medical Laboratory, College of Health Sciences, Woldia University, Woldia, Ethiopia; 6 Department of Public health, College of Health Sciences, Injibara University, Injibara, Ethiopia; 7 Department of Public health, College of Health Sciences, Debre Markos University, Debre Markos, Ethiopia; 8 School of Public Health, College of Health Sciences, Woldia University, Woldia, Ethiopia

**Keywords:** Armed conflict, Health consequence, Coping mechanisms, Qualitative study, Ethiopia

## Abstract

**Background:**

Conflict is a complicated topic with a multidimensional consequences for community health. Its effects have a broad pattern, starting from direct war-related morbidity and mortality caused by bullets and bombs to indirect consequences due to the interruption of the delivery of preventive and curative health services. This study aimed to explore the health consequences of the northern Ethiopian conflict in the North Wollo zone, northeast Ethiopia, in 2022.

**Methods:**

This descriptive qualitative study was conducted from May to June 2022 on six conflict-affected Woredas in the north Wollo zone. A total of 100 purposively selected participants, which included patients, pregnant women, elders, community and religious leaders, and health professionals, were interviewed using IDI and FGD. The data was entered, coded, and analyzed using Open Code version 4.03. Thematic analysis approach employed to conduct the interpretation. Data was presented using descriptive statistics in the form of texts and tables.

**Results:**

The findings indicate that the conflict has caused a profound consequence on population health. It has resulted in a wide range of direct and indirect consequences, ranging from war-related casualties, famine, and disruptions of supply chains and forced displacement to instances of violence and rape associated with insecurity. The conflict also caused a breakdown in the health system by causing distraction of health infrastructure, fleeing of health workers and shortage of medication, together with insecurity and lack of transportation, which greatly affected the provision and utilization of health services. Additionally, the conflict has resulted in long-term consequences, such as the destruction of health facilities, interruption of immunization services, posttraumatic stress disorders, and lifelong disabilities. The coping strategies utilized were using available traditional medicines and home remedies, obtaining medications from conflict-unaffected areas, and implementing home-to-home healthcare services using available supplies.

**Conclusion:**

The Northern Ethiopian conflict has an impact on community health both directly and indirectly through conflict-related causalities and the breakdown of the health system and health-supporting structures. Therefore, this study recommends immediate rehabilitation interventions for damaged health infrastructure and affected individuals.

## Background

The tragedy and constricted northern Ethiopia conflict started in early November 2020 when Tigray People Liberation Front (TPLF) forces attacked the federal military bases in the region [1, 2]. Since then, the war continued in the Tigray regional state for two years and in late June 2021, the war expanded to the neighboring region of Afar and Amara, where many atrocities to civilians and infrastructures were reported [3–5].

Armed conflict has profound implications for public health, giving rise to a diverse array of consequences that can be categorized as either direct or indirect. The direct consequences primarily encompass the immediate morbidity and mortality resulting from the utilization of firearms and explosives during conflict. Evidence indicates that casualties arising from conflict-related incidents often exceed the rates of morbidity and mortality typically observed in areas unaffected by conflict, impacting both military personnel and non-combatant civilians.

 [6–9] [6]. The World Health Organization (WHO) estimated that in 2002 alone, war resulted in the deaths of 172,000 individuals worldwide [10]. Additionally, Burnham et al. reported 601,000 deaths attributed to violence during the Iraq war, spanning from March 2003 to July 2006. Moreover, the same study found a significant increase in the crude mortality rate during the conflict, surpassing the previous rate by more than twofold (from 5.5 deaths per 1000 people to 13.3 deaths per 1000 people [11].

In addition to the direct consequence, conflict also indirectly deteriorates the health of the population by causing breakdown of the health system, shortage of medical supplies and displacement of healthcare workers, as well as disruption of food and clean water supplies. Furthermore, conflict-related insecurity and a lack of free movement also reduce the provision and utilization of health services, with patients hesitating to seek healthcare due to concerns about their safety or potential targeting when traveling to healthcare facilities [12–14].

A study conducted in the Zapatista conflict zone of Mexico reported that only 6.9% of labouring mother delivered in health facilities [15]. Likewise, a study conducted in Syria showed that the air bombardment and explosions reduced the odds of maternal service utilization in conflict affected areas [16].

Despite these significant consequences, the health impact of conflict is still poorly addressed due to the breakdown of health information systems, particularly civil registration systems that record events and causes of death [6, 7, 17]. Additionally, available information may also be politicized and intentionally misrepresented [6, 17]. For this reason, the WHO passed a resolution in 2012 that calls for leadership in documenting evidence of attacks against health workers, facilities, and patients in armed conflict settings [18].

Concurrently, understanding the health impact and consequences of armed conflict in post conflict settings present a window of opportunity, to develop responsive and evidence-informed strategies for addressing the defects in community health [19, 20].

In the northern Ethiopia conflict, there have been numerous reports of destruction and atrocities against health systems and civilians [3–5]. However, most of the reports have relied on press reports of eyewitness accounts, journalists, humanitarian agencies and official announcements of combatants. To the best of our knowledge, there is no previous study on the area, that clearly demonstrates the consequences of the conflict on the health and health system, particularly from the perspective of affected individuals and communities.

Therefore, this study aims to explore the health consequences of the northern Ethiopia conflict with the aim of highlighting the burden of these problems from the perspective of affected individuals and communities. The findings of this study will provide insight for humanitarian interventions and serve as input for government and other stakeholders in designing evidence-based health interventions for conflict-affected areas. It will also serve as baseline evidence for further investigation in the area.

## Method

### Study area and period

A retrospective qualitative study was conducted on conflict-affected areas of the North Wollo zone of Amhara regional state to explore the health consequences of the northern Ethiopia conflict during the six-month period of TPLF occupation of the area. The area was under the control of TPLF fighters for about six months, from July to December 2021.

The North Wollo Zone is one of 10 zones of the Amhara region located about 521 km from Addis Aba, the capital city of Ethiopia. It is bordered by the Tigray Region in the north where the conflict started and was confined for 2 years until it broadened to the zone. The zone has 14 Woredas and 313 Kebeles with a total population of 1,500,303 and 355,974 households. The study was conducted from May to June 2022 GC. We followed a phenomenological approach to understand and describe the health challenges and experiences of the community during the conflict.

### Sampling and study participants

The sampling technique employed in this study was a multistage sampling. First, from the 14 Woredas in the north Wollo zone, Guba Lafto, Habru, Kobo, Lasta, Wadla and Woldia Woredas, were selected randomly by the lottery method. Next from each selected Woredas, one kebele was selected randomly. Finally, information was collected until the data was saturated from purposively selected key informants living in the selected Kebeles.

The key informants were purposefully selected based on two main criteria. The first criterion was first-hand experience, which included individuals who were directly affected by the conflict, such as those who were sick or had sick family members during the conflict, chronic patients, and pregnant mothers. The second criterion was knowledge and exposure, which included individuals who had a better understanding of the health condition of the community during the conflict and had more exposure to affected individuals. This category included health professionals, community and religious leaders, and members of different community associations, including the peace committee and Edir. By selecting key informants who met these criteria, we aimed to gain a comprehensive understanding of the health situation during the conflict from different perspectives. Individuals who are below the age of 18 and who fled the area during the time of invasion and returned after the area was liberated were excluded from the study.

### Data collection method and procedures

The data was collected in the local language (Amharic) by three authors separately using one trained health extension worker who works in the respective areas as assistance. As the location was in a post-conflict area, to avoid incidents that may affect the safety of the data collectors, before starting data collection, information about the study, was provided to the public in community gatherings such as churches and mosques. This approach promotes transparency, builds trust and encourages community participation by providing an understanding of the research objective. In addition, to avoid the recall bias associated with the retrospective study, a semi structured interview guide was used for in-depth interviews and focus group discussions. The questions were open ended with individually adapted follow-up probing questions. Introductory questions asked for basic personal data, including age, educational level, and residence. Each conversation was recorded, and field notes were taken to supplement the recordings. Information was collected until the data was saturated. In total, 40 in-depth interviews and 6 focus group discussions (each with 10 respondents) were conducted with key informants. The interviews and FGDs lasted for 30 to 120 min.

#### Issues discussed

The interviews and FGDs focused mainly on exploring the health consequences of the northern Ethiopia conflict, the process assessing the health condition of the community during the conflict and the mechanism through which the conflict affected the health of the community. The issues discussed include; the health status of the community during the conflict, the mechanism through which conflict affects the health of the community, the effect of conflict on the health and health system, the most affected groups of the community, the coping mechanisms utilized for the health challenges of the conflict, and the long-term health consequences of the conflict.

### Data analysis

The data was audio-recorded and later transcribed by two investigators separately and translated into English. After comparing each translation and checking the consistency, the data was entered, coded and analyzed by three coauthors using open code version 4.03. Thematic analysis approach was employed to interpret the data. First, descriptions and specific ideas were coded separately, inter-related or similar codes were then grouped into different categories, and the categories were subsequently clustered into specific themes. Participant quotes were used to emphasize or support emerging themes in the result presentation.

## Result

### Socio-demographic characteristics

In this study, a total of 100 respondents in 40 in-depth interviews and 6 FGDs (10 individuals in each) participated: 27 ‘individuals who have been sick or have sick family members during the conflict’, 10’ pregnant women’, 14 ‘elders, community and religious leaders’, 14 ‘members of different community associations’, and 22 health professionals and health institution administrators. The focus groups comprised at least one representative from each key informant for the 5 FGDs, with the exception of health professionals and health institution administrators, who solely made up the remaining one FGD (Table 1).

**Table 1:** Composition and characteristics of the study participant in exploring the health consequences of armed conflict in Northeast Ethiopia, 2022 (n = 100)

S.no	Types of Respondent	Focus group discussion	In-depth interview	Total
1.	Individuals who have been sick or have sick family member during the conflict	15	12	27
2.	Chronic patients	5	4	9
3.	Elders, community and religious leaders	10	4	14
4.	Member of different community associations	10	4	14
5.	Health care professionals	8	9	17
6.	Health institution administrators	2	3	5
7.	Pregnant woman	10	4	14
8.	Total			100

The mean age of the respondents was 36 (SD ± 11), and half of them were in the age group of 30–44 years. Most of the respondents were married (77%). Additionally, more than half of the participants were urban residents (56%) (Table 2).

**Table 2:** Socio-demographic characteristics of the study participants in exploring the health consequences of armed conflict in Northeast Ethiopia, 2022 (n = 100)

Variables		Frequency	Percentage (%)
Age	< 30	25	25
	30–44	52	52
	≥ 45	23	23
Sex	Female	48	58
	Male	52	42
Residence	Urban	56	56
	Rural	44	44
Marital status	Married	77	77
	Single	11	11
	Divorced	7	7
	Windowed	5	5
Educational status	No formal education	24	24
	Primary education	31	31
	High school and preparatory	16	16
	College and above	29	29

### Health status of the community during the Northern Ethiopian conflict

The study shows that the conflict had inflicted a grim consequence to the health condition of the community, affecting individuals, families, and entire communities, with lives torn apart and supply chains disrupted.

A 60-years-old participant who was a member of the peace committee explained the situation, as follows: *“It is difficult to mention the hardship we passed. It was like living in a dark room without the knowledge of where to go. There was no water for drinking or electricity for cooking. There was no food to eat, as there were no millhouses and no market days, and the price was beyond the capacity of the community to pay.*

The community was in difficult health condition with respondents reporting a high number of morbidities and mortalities during the conflict. One of the most reported consequences of conflict was the direct consequences associated with conflict causalities, which included gunshot wounds, bomb blasts, and other forms of violence, that resulted in debilitating injuries, disability and death.

*“Everything appears to be like a dream. Each day civilians lose their lives due to lack of food, lack of medicine and gunshot trauma. Many have also become disabled and are now living a miserable life.” (Said one community elder)*.

Additionally, the conflict has resulted in the disruption of healthcare systems which coupled with the breakdown of supply chains, has caused a profound increase in disease-related morbidity and mortality.

### Mechanisms through which the conflict affected community health

Generally, we have thematized the several mechanisms through which the conflict affected the community’s health, such as conflict related causalities, famine and food shortage, displacement, insecurity, and lack of health services.

#### Conflict related casualties

The injuries, trauma, and loss of life inflicted by the conflict-related gunshots and artillery shooting, were reported as a main factor contributing to the health crisis, exacerbating the community’s health situation. One health extension worker in one of the conflict front areas recounted this as follows;

*“…each day, civilians had lost their lives due to gunshots. Heavy artillery has attacked their homes. In our Kebele alone, there were 63 disease related and 45 conflict related deaths.”* She said.

This testimony further reinforces the findings that morbidity and mortality rates were substantially affected by both direct conflict-related factors and disease-related factors, painting a grim picture of the health situation during the conflict.

#### Famine and food insecurity

The conflict has indirectly affected community health by destroying supply chains and social support structures causing famine and food insecurity, which leads to hunger and malnutrition. This coupled with shortage of clean water and electricity has created a fertile ground for the spread of infectious diseases such as malaria, typhoid, dysentery, and respiratory infections, which have amplified the morbidity and mortality, associated with the conflict.

*“How can I express it?’* Said a hospital administrator of the tragic situation.*’ There was no problem that did not occur; some people developed typhoid fever by eating cold meals and drinking contaminated water from rivers and open sources, while many others died from famine.‘’*.

#### Displacement of the population

Conflict related violence and instability caused displacement of the population, which also greatly affected community health. Because most adults and family members have fled the area for fear of being targeted and forcefully recruited by the militants, the elders and people in need were left without supportive and caring persons.

*“I recall one elderly woman for whom I was called by neighbors to give care. When I arrived, I found that she had developed a bedsore in her back and was in critical condition. Later, I learned that she had been living alone for several days after her family fled the area. Due to this, there was no one to help her when she became sick and bedridden.”* (Said one nurse).

#### Insecurity and Violence

In conflict settings, the lack of protection from government and civil institutions resulted in a high level of sexual violence, with soldiers and civilians using rape to humiliate and spread fear among the community[40, 58, 59]. There were many reports of sexual assaults and rapes of women and adolescents in the current study, with serious health consequences for the survivor. The wife and daughters of solders were particularly targeted as a strategy to humiliate the solders and their family.

*“It was terrifying. Particularly, if they know you are a military family they will come and assault you, I know a woman in our neighborhood who has husband in the military; the Juntas come to her house in broad daylight and rape her in front of her 5-year-old child (scared tone while crying).”* (Said the survivor’s neighbor).

#### Lack of health services

Moreover, due to a lack of functional health services provision, and associated medication shortages, patients were taking inappropriate and lower-quality medications.

*“One of the burning health issues we had was the usage of unprescribed and expired medications. Because there were no pharmacies or medical supplies, patients were taking whatever medications they could got without checking the prescription or expiration date. For example, if they become sick due to pneumonia and find a typhoid medication in their house, they will take it.‘’* (A health center manager said).

### The devastating consequences of conflict on the health system

The poor health condition of the community was exacerbated by the health system’s disruption, which ranged from the partial or complete shutdown of medical institutions to shortage of health workers and medical supplies. The complex and multifaceted way through which the conflict affected the health system have been thematized as the following; closure of health facilities, shortage of medication and supplies, displacement of health professionals, insecurity and lack of free movement, and overtaking of health facilities by combatants.

#### Closure of health facilities

The conflict has caused closure of most of the health facilities in the area; only two hospitals and three health centers were the only health facilities that were providing service. However, due to resource shortages there services were limited. This was caused by fear of being targeted, displacement of health professionals and shortage of supplies. The closure of the facilities also places an enormous burden on the functioning institutions.

*‘During that period, obtaining healthcare was a challenging task for the community. The only hospitals available in the area were Woldia Hospital and our (Lalibela) Hospital, but their ability to provide services was hindered by a shortage of medical professionals and medications. As a result, they were merely supporting those with emergency.” (Nurse at Lalibela hospital)*.

Not only did the conflict disrupt public healthcare facilities, but it also affected the private healthcare system. Private clinics, pharmacies, and laboratories have been forced to close. In certain areas, the TPLF fighters were accused of forcing the private service providers to close their facilities.

*’The closure of private pharmacies and clinics exacerbated the problem. Who will have the courage to open his clinic at such time? All have closed and fled the area; those that remain have no medications, and even if they did, they would not open because they fear for their safety. For example, there was one doctor who had a private clinic, and every time he tried to provide service, the Juntas would go and ask him for money; eventually, he was forced to close his clinic and fled the area.”* (A 32 year old male nurse with 5 years of experience).

#### Displacement of health workers

It also resulted in the forceful displacement of health workers, with many fleeing the area due to security concerns.

*‘’It was difficult to find health professionals and other health workers at the time because they had fled the area due to security concerns; only a few remained, and some have yet to return.”* (Said a health center administrator).

#### Shortage of medication and supplies

The conflict has caused disruption in the supply networks of health facilities, leading to widespread looting of medical supplies, and scarcity of pharmaceuticals.

*‘’Following the government troops left the area; there was no one to gourd the hospital. This coupled with concern about potential medication shortages under the control of the juntas (opposition fighters), led individuals to ta*k*e advantage of the situation and engage in* loot*ing* the medications. Up on the Juntas’ arrival, they also took the remaining medication.‘’ (Said one pharmacist).

This shortage of medication limits people’s access to essential healthcare services, especially for those with severe disease. This can result in delayed or inadequate treatment, increased morbidity and mortality rates, and a decline in overall health outcomes.

*“My friend was sick, so we took him to the health center. When we finally arrived there after much struggle, they informed us that he had kidney disease and that there was no available medication to treat him. Instead, we were advised to go to the hospital in the hopes of a better outcome. Unfortunately, when we arrived at the hospital, the situation remained unchanged, leaving us empty-handed;“* A 45 year-old resident.

#### Insecurity and lack of transportation

In addition to the shortage of professionals and supplies, the widespread instability and lack of transportation have had a significant impact on health provision and utilization, affecting both the health workers and patients.

*“Even if we become ill we will not go to health institutions. Going to health facilities was difficult; there was a security check everywhere; the Juntas will stop you and check you; if you’re lucky, they will take your money for treatment, your phone, everything, and let you go; if not, you might be beaten or take you away”. Furthermore, the absence of electricity and the restriction of movement at night further complicated the issue. Consequently, healthcare services could only be accessed between 6:00 a.m. and 6:00 p.m.” (A 25-year old male* resident*)*.

Health providers have also faced an enormous challenge in going to health facilities, because travel at night was very unsafe, with harassment, assault, or extortion by armed persons at roadblocks a common phenomenon.

*“We the health professional, were facing many challenges. First, due to the absence of transportation services in the town, we had to undertake a long journey on foot to reach the hospital. Second, the prevailing insecurity was highly frightening. There was no any protection for the health workers, and no one will take responsibility for ensuring your safety. We were coming with terror, especially at night; many people have been robbed while coming to the hospital.”* (28-year old female anesthetics with 4 years of experience).

#### Overtaking of health facilities by combatants

Some health facilities were also forced to use their limited medications and supplies for treating the wounded fighters. ‘*‘When the fighting becomes intense, they bring their injured fighters to the hospital and use it as a shelter to protect themselves from drone strikes. They also threatened to take over the hospital and turn it into a military camp if we did not treat them.”* (A 32-year old medical director).

Some were even taken over by the combatants. This undermines their impartiality and compromises the perception of the community. As a result, even if the fighters were providing service through their own personnel, the community was not using the service.

*‘’After they took over the hospital, they told us to go and get treatment, but who would have the courage? Who knows what they will provide? What if they poison us? Off cures there are some severely ill patients and laboring mothers who have go there and get treatment, saying if we die lets die there.”(A 67-year old priest)*.

This finding indicated that although the conflict had impacted nearly all segments of the community, vulnerable individuals, including those with chronic disease, pregnant mothers, and children, experienced greater suffering due to the absence of routine care and follow-ups.

#### Chronic patients

During humanitarian emergences such as conflict, people living with chronic disease such as hypertension, diabetes, cardiac diseases, and HIV become more vulnerable due to limited access to essential health services. This vulnerability is also evident in the northern Ethiopia conflict, where many individuals with chronic disease have been suffering due to a lack of treatment and follow-up care.

*“The conflict period was very difficult, especially for individuals with chronic illness who relied on regular medication and follow-up. They were suffering due to a lack of medication, and tragically, many lost their lives.”* (Nurse with 22 years of experience).

*“I was very ill, and my medication was running out. This, combined with the famine puts me in a stressed and desperate position. Later, I lost all hope and began to wonder, “What is the meaning of life?“ “I even made numerous attempts to end my own life, knowing it was a sin for which I would have to repent with my soul father later.“* (Said a 65 year old HIV patient).

Being pregnant and giving birth involved a particularly vulnerable position in setting of conflict. Pregnant women in conflict settings encounter considerable challenges during and after childbirth, with limited possibilities of accessing quality intrapartum and postpartum care, and a lack of adequate nutrition and support after childbirth. In the current study, almost all respondents from all areas expressed that pregnant women were among the most affected groups of the community during the conflict, due to lack of access to perinatal and delivery services.

*“Labouring woman were giving birth at home without the assistance of a skilled provider. They didn’t even get the help of a traditional birth attendant, as it had already become a trend to give birth in health facilities.”* (Said one midwife).

Pregnant women and their children were also suffering from the prevailing food insecurity and famine, which exposed them to risk of malnutrition and its associated complications.

*“It was tough to find something to eat for a new mother who needs a lot of food to recover. There was no market day, and even if there was, the price was unaffordable with double or triple of the regular days. For instance, milling half a quintal of grain would cost around 800 Ethiopian birr.”*.

In addition, as one health extension worker recounted, labouring mothers and their babies were also subjected to traditional malpractice, which increased their risk of childbirth related morbidity and mortality.

*‘’I remember a specific incident where I was called by the relatives of a mother who required my assistance. She was bleeding after giving birth for a prolonged labour. Since the area was far, by the time I arrived, the baby had already passed away, and the mother was experiencing heavy bleeding with the placenta retained. Later, I discovered that while I was on my way, they attempted to remove the placenta by vigorously shaking and rotating her upside down multiple times.‘’* She said.

Furthermore, because of the disruption of the provision of disease preventive and health promotive services, there has been a spike in both vaccine-preventable and nonpreventable diseases in children.

*“One of the burning health problems we were facing was the highly prevalent child illness. Every day, numerous children come for the treatment of diseases such as diarrhea, trachoma, and pneumonia. However, many of them would return empty-handed and would have to wait for many days without getting any treatment due to a shortage of medication and health care professionals.”* (One health center administrator said).

## Conflict elicited health challenges and coping mechanisms utilized that enhanced resilience

The conflict gave rise to the abovementioned and other various health challenges, which profoundly affected the health of individuals and the community. In response to these challenges, people utilized different coping mechanisms to navigate through the difficulties they faced. These coping mechanisms were diverse and varied depending on the individuals involved. Overall, three subthemes emerged from the study: [1] coping mechanisms utilized by patients; [2] community cooperation and religious leader’s roles as coping mechanisms; and [3] coping mechanisms utilized by health care providers.

### Coping mechanisms utilized by patients

The conflict-elicited breakdown in the health system caused a huge challenge for patients, as they were unable to access necessary treatment and medications for their illnesses. To mitigate this challenge, patients have employed various coping mechanisms, with the most commonly, reported strategies being the use of traditional medicine and home remedies, followed by traveling to areas unaffected by the conflict in search of treatment and medication.

*During that time, you would better pray to not get sick because if you did, there was nothing you could do. Your only option was to use traditional medicines and home remedies. Even doing Dua (praying) together, was not possible as gathering more than three people was prohibited.”* (Said one religious leader).

Some patients have also attempted to seek treatment and medications by traveling to unoccupied areas. However, this was also challenged by the insecurity and a lack of transportation.

*“When they fell ill, some patients have attempted to go to unoccupied areas in order to receive treatment. However, due to fear of being captured by the Juntas, they had no choice but to traverse the desert on foot. Consequently, only those who had the physical strength or had someone to support them were able to undertake the journey.“* (Said one HIV patient).

Even if they were able to reach those areas, as they often would go after their situation becomes complicated, many individuals have died along the journey. *“I remember a tragic incident where a 16-year-old child, who was traveling with his father and had passed away during their journey. It was terrible.‘’* Said another respondent.

### Community cooperation and religious leaders’ role as coping mechanisms

The community has tried to alleviate these challenges and problems, using different mechanisms and platforms. The main mechanism was the cooperation and support of community members with each other. The formation of peace committees consisting of elders and members of the community is an excellent example of community cooperation in addressing conflict elicited health challenges and problems. Such committees played a crucial role in fostering unity, helping those in need, protecting health facilities, and promoting overall well-being.

*“When my wife was suffering from severe hypertension and was on the verge of death due to a lack of medication, our neighbor, who had a similar illness, obtained some medication from her relatives and shared it with her. She said ‘if we die, let us use it and die together.’ She survived using that medication until our father brought medication from Bahr Dar and gave it to us for free.”* (Said one respondent).

*“*The community’s cooperation was very good, as people voluntarily shared the limited food they had with those in need. Personally, I made an effort to contribute by collecting food from the community and distributing 3 kilograms of grain flour to the most needy, advising them to use it as MUQ or ATMIT (a local soft food made by boiling flour of wheat or legumes in water until it is thick) to save food.*”* (A 50-year-old community leader said).

The other main buffer for alleviating the health consequences was the exemplary role of religious leaders. Particularly, in Woldia and Lalibela, the religious leaders played a significant role in stabilizing the community and organizing health facilities.

*“The role of our father (religious leader), was significant in our survival. He was the one who helped us through this difficult time. He collected cash and foods from each person in the name of the church and provided for those in need. He also gathered and encouraged the available health professionals to serve the community. At the same time, to ensure they will not be hungry while serving the community, he personally provides them with cash and food, even taking the initiative to cook it himself.”* (A 45-year-old member of peace committee).

In some areas, religious leaders in collaboration with the peace committee also brought medications and supplies from unoccupied areas using the available routes.

‘*‘When our medication was completely depleted and numerous patients were facing death due to the lack of medications, the religious leaders and peace committees communicated with people in Bahr Dar and brought medications using animal power. This saved the lives of many individuals, especially those of chronic patients, who were in urgent need of medications.”* (One medical doctor said).

*“When I was feeling hopeless and in disparate situation, our fathers brought medications and gave them to us free.” (A 62-year old diabetic patient)*.

In addition, religious leaders encouraged patients who were able to obtain medications through various means to share them with others facing similar conditions. At one point, the priest even went as far as giving his own medications to those in need.

*“When the people were suffering due to a shortage of medication, our father said one day while the people were gathered in the church, ‘I am already old, and there is nothing more I can do. However, if they survive this horrible time and reach tomorrow, the others are the hopes of this country. Therefore, please take the remaining of my medication and give it to those in need. It may save at least one person.’ This heartfelt plea greatly inspired the community, motivating them to share their medications with one another.”* (A 45-year old health center administrator).

Moreover, the active involvement and support of religious leaders serves to inspire the community and individuals, fostering a strong sense of hope and faith in their religion. This enables them to cope with feelings of despair and stress, while simultaneously encouraging mutual support in difficult situations. They repeatedly invoked “the will of God” and articulated religious beliefs to make meaning of their circumstances and cooperation.

*“When we witnessed our father doing everything, while he could have fled the area, we started to have hope. We know God, who gives us a father like him, will not fail us. Who knows what would have happened if God had not given us a father like him.”* (A 55-year old Edir leader).

Our findings also reported that in addition to serving as coping strategies for the community, religious leaders’ encouragement was also important source of motivation for health workers. It encourages the health workers to leverage religious beliefs and feelings of duty to provide services.

*“When you see our father traveling up and down on foot, to collect food and money from those who have, and personally cooking to provide for health workers and those in need, you would be very encouraged. Such selfless dedication motivates you to set aside personal concern and do everything you could to support those facing in difficulties.”* (Said one midwife).

Religious leaders have also played a major role in protecting the health facilities. *“When they start to bring and accumulate their injured fighters to the hospital, his holiness said to them, ‘this is my people’s hospital, not your military hospital, stop bringing your solders here. If not, open the road for me. I will take my people, and you can be as you want afterward.”’* (Said one medical director).

### Coping mechanisms utilized by health workers

This study demonstrates that health workers were at the forefront of effort to cope with the devastating health consequences of the conflict. They exerted maximum effort to mitigate the health challenges faced by the community. Even disregarding their own safety. Despite the insecurity and limited freedom of movement, which hindered the community’s access to health facilities, the health workers divided themselves and served their respective neighborhoods by conducting house-to-house visits. Their dedication and commitment to providing healthcare in challenging circumstances played a crucial role in supporting community well-being.

*“Since most of the health facilities were closed, we were providing home-to-home services in our respective areas. In our kebele, one gynecologist and me were the only available health professionals. We did our best to serve as much as we could. Even during the night, when there were emergency cases such as laboring mothers, families would come and fetch us. I even had confrontation with my husband because he was concerned for my safety when I went out at night. However, if I do not go, they might die. By doing so, we were able to save many lives. Personally, I assisted around nine labouring mothers alone.”* (Said a health extension worker with 9 years of experience).

Additionally, when facing shortage of materials and supplies, the health professionals worked with the available materials they could obtained.

*‘’When we were providing home-to-home service, one of the main challenges we were facing was lack of materials and supplies. Mainly, during the delivery of labouring mothers, we encountered a lack of surgical gloves, and suturing stiches. Our only option at that point was using plastics as surgical gloves and cloth stitches as surgical stitches.”* (Said one midwife).

Furthermore, to prevent the onset and spread of sporadic diseases resulting from the usage of unclean and surface water; health professionals took the initiative to provide health education. They emphasized the importance of boiling water before use and maintaining personal hygiene practices. By raising awareness about these preventive measures, they attempted to minimize the risk of waterborne disease.

*‘’When the outbreak of diarrhea and typhoid began, each professional took the initiative to educate the community in their respective neighborhood about how to clean the water and keep personal hygiene”* (Medical director).

## Long term health impact of the conflict

The invasion has resulted in numerous direct and indirect health consequences that will have long-term impacts on the well-being of individuals and the community as whole. Among the consequences are the mental health problems experienced by individuals and the collective community, with many developing psychological trauma. Additionally, the conflict has exposed children and adolescents to destruction, atrocities, and life threating situations, which can have devastating impacts on their wellbeing throughout their lives. This was best described by a 70 -year-old priest as follows:

“The community’s psychology has been deeply damaged, and it will not easily heal. They entered each house and humiliated us. We have seen our children being raped in front of their parents. They undressed women in front of their families. Men were forcibly undressed and checked in their homes. How can an individual who has witnessed such an unimaginable act live their life as usual? We have untreated scare. Even now there are people who become anxious when they see a person with military uniform.”

The conflict also caused a significant destruction of health facilities that will require many years of effort to rebuild and restore. The loss of these facilities can have long-lasting effects on the accessibility and quality of healthcare services available to the community, creating additional barriers to achieving optimal health outcomes. Furthermore, the extensive damage poses a significant challenge to post-rehabilitation efforts and the future provision of health services, further undermining the overall health and well-being of the community.

*“Our health post was once well equipped by UNICIEF, but now it stands empty. There is no equipment, and we are even buying documenting materials out of our pockets.”* (A health extension worker explained).

Additionally, the conflict resulted in a significant amount of sexual violence and rape, leaving the survivors with long-term physical, medical and psychological complications. The survivors of these heinous acts endure immense suffering and may face ongoing health challenges because of their traumatic experiences.

*“I witnessed a girl being shot because she refused to be raped. She is still in the hospital receiving treatment. Many raped women have acquired HIV and become pregnant from their perpetrators. I couldn’t explain what they would feel.”* (One medical doctor reported).

Furthermore, the conflict has disrupted the delivery of health promotive and preventive services including, immunization and PMTCT programs. As a result, its full impact has yet to be determined. This is noteworthy, as the interruption of immunization services can have far-reaching consequences on the community’s overall health, leaving individuals vulnerable to vaccine-preventable diseases and potentially affecting long-term health outcomes.

*“Since there was a lack of ART medications, HIV positive pregnant mothers were unable to receive PMTC services. Some of them even would come to us, and request ART medications, but unfortunately, we would send them empty-handed, by only advising them to seek from other unoccupied places if they had the means to do so. For this reason, I fear that the number of children born with HIV would have been considerably high.”* (Said a 46 year old midwife).

## Discussion

This article contributes to a more holistic understanding of the extent and range of conflict effects on community health. It provided a deeper understanding of the health experience of the community during the conflict. It also sheds light on the complex health challenges faced by patients and the community, with the coping strategies implemented to attenuate these challenges.

Consistent with other studies conducted in conflict settings, this qualitative study revealed that, amid violence and insecurity, the health condition of the community was greatly compromised during the northern Ethiopia conflict [11, 21, 22]. This was exemplified by a high number of both violent related and disease related morbidities and mortality in the community reported by the participant [25]. [12] [26–28]These can be understood as part of a broader pattern impact of conflict where direct killing of civilians by armed personals, artilleries and armed drones’ shelling become the distinguishing feature of several recent armed conflicts [12].

The conflict also caused tremendous consequences to health of the community in indirect ways including, the lack of electric and clean water supplies, which resulted in influx of communicable, and outbreak diseases such as typhoid and malaria. Similarly, the conflict inflicted famine and food insecurity that resulted in an increased number of individuals and children affected by malnutrition. These consequences were mainly evidenced in susceptible individuals including; chronic patients, elders, pregnant mothers and children. This was in line with other reports in similar settings, where conflict related disruption of the health system affected vulnerable individuals who need continuous care and follow up [22–25].

The conflict also affects the health of the community by inflicting collapse in the health system, causing closure and looting of health facilities, displacement of health workers and disruption of the supply chain. Only two hospitals and three health centers were providing health services at that time. This shows that the health service provision in the area was far behind the minimum standards required for health service delivery as set out by relevant guidance such as the Sphere Guidelines on the Humanitarian Charter and Minimum Standards in Disaster Response and the UNHCR emergency handbook [26, 27]. This has greatly limited the community’s access to and utilization of health services. This finding is in line with studies conducted in Uganda, Liberia, and Syria, that reported a negative link between conflict and the utilization of health services [15, 16, 28, 29]. In this particular study, despite some reports of health-care facility destruction, there were no reports of systematically targeted attacks or bombings of health-care facilities, which unfortunately were an all too common occurrences in many other conflict zones and have become a troubling trend in recent warfare [30, 31]. As an example, the International Committee of the Red Cross (ICRC) recorded a total of 921 instances of violence directed towards healthcare services in 22 countries undergoing armed conflicts and emergencies in 2012. This difference could potentially be attributed to the influence of the peace committees and religious leaders in the current conflict. It might also be partly related to the war primarily taking place in rural areas, while most healthcare facilities were situated in towns, which reduced the likelihood of deliberate attacks and bombings targeting them.

According to the study, the conflict not only disrupted public health services but also caused a great breakdown in the private health system, causing forceful closure of many private clinics and pharmacies. This was in contradiction with a previous study conducted in Somalia, where the private health care filled the vacuum generated by the absence of public health care [32]. It was also in contrast to other areas, where the multiplicities of private health actors resulted in further deterioration of the public health system [33].

In responding to these complex challenges, patients, health workers and the community have illustrated a variety of problem-focused coping strategies. The main coping strategies utilized by patients were the use of home remedies and traditional medicines, and traveling to war non-affected area to obtain treatment and medications.

Likewise, health workers have employed innovative measures to cope with the conflict-associated closure of health facilities and the dearth of supplies. This includes; the provision of health services at the community level and uses of available materials such as; plastics as surgical gloves and cloth stitches as suturing materials. Similar coping strategies were highlighted in the context of the Syrian crisis, whereby health workers utilized different methods to cope with the shortage of medical supplies [34].

Furthermore, the community cooperation and support of religious leaders and volunteer peace committees have played a pivotal role in stabilizing the area and limiting the health consequences. This report shares similarity with findings reported in certain parts of Africa, where the church and its leaders have played active roles in stabilizing conflict settings [35]. The active participation of community and religious leaders has proven to be a significant force, enabling individuals and communities not only to maintain their faith but also to draw strength from their religious beliefs to endure the immense hardships they face during armed conflicts. This involvement has helped them deal with overwhelming feelings of despair and stress, while also fostering a supportive environment in challenging circumstances. Similar findings were reported in other studies where communities impacted by conflict employ religion and social support as means to strengthen their resilience and manage the psychological distress linked to such conflicts [36–38].

The results also revealed that the conflict has inflicted many lifelong disabilities secondary to bullets shots, and psychological trauma in those who have experienced destruction, atrocities, and threats to life, leaving a devastating impacts on their wellbeing throughout their lives. A similar finding was reported in a previous study done among young Liberian refuges where participants reported, how the scenes of abductions, killings, seeing dead bodies, and wounded people constantly haunted them in their lives [39, 40]. Consistent with previous studies the current study also showed that, the conflict disrupted the provision of many health promotion and preventive services including immunization and PMTCT; hence, its full impact is yet to be determined [41, 42]. Furthermore, the conflict has caused a huge destruction of health facilities, which would take many years and effort to reestablish and reorganize; jeopardizing community health for the long term.

### Limitations of the study

It is important to consider these findings in light of their limitations. First, the data was collected six months later, after the government had taken control of the area. As a result, there might be a recall bias. Second, our data only capture the perspectives of people who stayed in the area, leaving out the health experiences of displaced peoples, for whom the conflict may have had just as many, if not more, health consequences. Third, the study did not assess the psychological and mental health issues individuals have experienced during the conflict due to the difficulties in diagnosing these issues based on retrospective reports from participants. Therefore, caution must be exercised when applying these findings.

## Conclusion

In conclusion, the findings in this study show that the Northern Ethiopia conflict has a multitude of consequences on the health of the community. These consequents were caused by both direct war related causalities and indirect break down in the health system and health supporting structures. The study also showed that the conflict have caused disruption of health promotive interventions together with lifelong disabilities and posttraumatic disorder which could have a long-term health impact.

To alleviate this issue, Ethiopia’s minister of health and other health actors who work in post conflict settings, should develop strategies aimed at tackling both the short term and long-term impacts of the conflict. These strategies should focus on reorganizing destructed facilities, providing rehabilitation programs for affected individuals such as survivors of violence and establishing vaccination programs for children’s borne during the time of conflict. There is also a need to develop a testing and treatment strategy for individuals affected by sexual violence related STIs, such as HIV, and for pregnant mothers and their babies who have not received PMTCT services.

The findings of the study also shed lights on the coping strategies utilized to alleviate the broad consequences of conflict that could be learned to other similar settings to support the resilience of communities affected by conflict. In particular, the role of religious leaders and community cooperation in both stabilizing the community and minimizing the health consequences of armed conflict deserves to be learned.

Further well-conducted studies are also needed to add weight to the conclusions drawn so far. In particular, on the experiences of chronic patients and pregnant mothers were found to be the groups of individuals most affected by the conflict. Further efforts should also be made to assess the experience of survivors of violence and its health impact on displaced people, who were not included in the study. Nonetheless, the findings of the current study should be borne in mind when performing postconflict reconstructing and rehabilitation programs.

## Data Availability

Data cannot be shared publicly due to ethical considerations, but could be accessed from the author with the request of Woldia University Research and Community Service Office/Woldia, Ethiopia (kingyimer@yahoo.com).

## Abbreviations/Acronyms

ART: Anti Retro Viral Therapy
FGD: Focus Group Discussion
HCWs: Health Care Workers
HIV: Humane Immune Virus
IDI: In-Depth Interviews
IRB: Institutional Review Board
MCH: maternal and child health
PMTCT: Prevention of Mother to Child Transmission
STIs: Sexually Transmitted Infections
SD: Standard Deviation
SPSS: Statically Package for Social Science
TPLF: Tigray Peoples Liberation Front
WHO: World Health Organization
